# Youth drinking and acute harm: a perspective from the UK on effective engagement opportunities

**DOI:** 10.1186/s13584-017-0165-0

**Published:** 2017-07-13

**Authors:** John Larsen

**Affiliations:** Drinkaware, Salisbury House, London Wall, EC2M 5QQ, London, UK

## Abstract

Youth drinking is linked with acute harms and Emergency Departments (ED) are places where some of these harms become apparent. This commentary argues that there is a clear case for routinely monitoring alcohol harm at ED and delivering brief interventions to engage with people at a time when they may be more likely to consider lifestyle behaviour change. Based on insights from the UK, it is suggested that young people may not always easily be engaged through messages that focus directly on alcohol harm, and efforts to engage them through issues that matter to them (such as sexual harassment) might add to the effectiveness.

## Commentary

The recent IJHPR article by Levinson and colleagues highlights issues of young people’s binge drinking linked to Emergency Department (ED) admissions in Israel, and presents a picture of alcohol harm that is familiar in a wider international context. An international study of patients attending thirty-seven EDs across eighteen countries found injury risk doubling at one drink and that injury levels differed according to country-level drinking pattern [1].

The survey on which Levinson and colleagues’ study is based provides useful snap-shot insights into the situation in Israel, being undertaken for a one week period and including two thirds of 16–35 year olds attending ED in a hospital in the Tel Aviv area. The finding that one in five of the women and one in four of the men were drinking several times a week shows that alcohol potentially may be a concern. However, in comparison, data from the Office for National Statistics show that in Great Britain in 2014, drinking rates were much higher; 48% of people aged 16–24 had consumed alcohol in the past week [2].

It would be helpful if future studies would clarify the extent to which the reason for attending the ED was directly related to alcohol consumption and harm resulting from this. Information on the extent to which the actual ED visit was directly related to alcohol harm could give further insight into the potential opportunity to engage the young people in a brief intervention related to this ‘critical event’.

In the UK, there has in recent years been increasing interest in using all relevant opportunities to offer brief interventions which offer advice on healthier lifestyle behaviours, known as the programme ‘Making Every Contact Count’ (see http://www.makingeverycontactcount.co.uk/). It is an approach to behaviour change that utilises the millions of day-to-day interactions that organisations and people have with other people to encourage changes such as stopping smoking, improving diet, increasing physical activity, losing weight and reducing alcohol consumption in order to reduce people’s risk of poor health.

As Levinson and colleagues suggest in their paper there might in Israel be an opportunity to be introducing a similar programme of brief interventions, and routine recording of information related to alcohol harm in the ED would be an essential aspect of such work.

When seeking to influence the behaviours of these young people it may however be important to consider the specific sociocultural meanings that are involved. In the UK the alcohol education charity Drinkaware in 2014 introduced a social marketing programme aiming at young people aged 18–24 who take part in the night time economy and self-report that they enjoy getting drunk. Qualitative insight research with this group highlighted that their behaviour is very much centred around the social activities of a night out and seeking adventure; and it suggested that they would not be motivated to reduce their drinking through messages highlighting the potential direct health harms.[3] Instead, the young people expressed concern around the more indirect consequences of drunken behaviours on a night out, and specifically the issue of sexual harassment was highlighted as unwanted, but the young people were finding it difficult to challenge this, as the behaviour was often considered to be ‘part of the game’ and as such it was silently tolerated. In response, Drinkaware developed a focus on messages to stigmatise and stand up against sexual harassment as part of a night out in the campaign ‘You wouldn’t do it sober, you shouldn’t do it drunk’ (see https://www.drinkaware.co.uk/advice/staying-safe-while-drinking/sexual-harassment/) and the in-venue intervention Drinkaware Crew supports individuals who may be vulnerable (see https://www.drinkaware.co.uk/about-us/our-campaigns/drinkaware-crew/). The programme of activity is currently running for a 3-year period in the North West of England and findings from the final evaluation will be available in 2018.

## Conclusions

The survey findings from an ED in Israel presented in the study by Levinson and colleagues provide some insights into the relevance of considering the impact of alcohol consumption on acute harms for young people. However, a better understanding of this would require routine monitoring in clinical practice. In order to effectively address the impact of alcohol consumption on acute harms there may be opportunities for a holistic public health approach that combines direct intervention at the individual level, using brief intervention techniques, with social marketing programmes. These could engage young people through issues that matter to them, aiming at shifting attitudes and behaviours linked to excessive and harmful drinking.

## Abbreviation

ED: Emergency Department
